# The effect of non‐pathological aging on behavior, genetics, and epigenetics in mice

**DOI:** 10.1002/alz.089172

**Published:** 2025-01-03

**Authors:** Sarah B. Scheinman, Bryan McClarty, Guadalupe Rodriguez, Gemma L. Carvill, Hongxin Dong

**Affiliations:** ^1^ Northwestern University Feinberg School of Medicine, Chicago, IL USA

## Abstract

**Background:**

Aging is a time‐dependent deterioration of physiological functions that occurs in both humans and animals. Within the brain, aging cells gradually become dysfunctional through a complex interplay of intrinsic and extrinsic factors, ultimately leading to behavioral deficits and enhanced risk of neurodegenerative diseases such as Alzheimer’s disease (AD). The characteristics of normal aging are distinct from those associated with age‐related diseases and it is important to understand the processes that contribute to this pathological divergence. The identification of behavioral, genetic, and epigenetic biomarkers associated with normal aging is key in determining the mechanisms of underlying pathological aging and how these impact AD.

**Method:**

We conducted a comprehensive behavioral assessment of young (3‐month‐old) and aged (18‐month‐old) C57/BL6 mice including locomotion, memory‐relevant, and anxiety‐like behavior to elucidate the cognitive and behavioral phenotypes of aging. We subsequently employed RT‐qPCR to determine synapse‐related gene expression in the prefrontal cortex of mice at both ages, and ChIP to assess age‐related differences in histone acetylation (H3K9ac) at the same genes. Finally, we conducted CUT&RUN sequencing to analyze the relative abundancy of H3K27ac, a histone marker that associates with promoters and enhancers of active genes to determine age‐related changes in epigenomic profiles.

**Result:**

Compared to young mice, aged mice displayed decreased locomotion in the open field (*p*<0.0001), enhanced anxiety in the elevated plus maze (*p* = 0.0003), and deficits in the novel object recognition task (*p* = 0.0011) and novel arm entry task (*p* = 0.0155). These age‐related behavioral changes occurred in tandem with lower abundancy of H3K9ac at synapse‐related gene promotors *nr2a* (*p* = 0.001), *glur1* (*p*<0.0001), *glur2* (*p*<0.0001), and *PSD‐95* (*p*<0.0001), and lower mRNA expression levels of *nr2a* (*p* = 0.028), *glur1* (*p* = 0.016), *glur2* (*p* = 0.038), and *PSD‐95* (*p* = 0.0037) in 18‐month‐old mice. CUT&RUN analysis also revealed significant age‐related differences in H3K27ac genome occupancy, including sites associated with synaptic plasticity and neuron function.

**Conclusion:**

These data suggest that non‐pathological aging induces a unique behavioral phenotype that’s associated with alterations in epigenetic markers and gene expression. Future studies will be focused on disentangling epigenetic and behavioral alterations that occur during normal aging from those that occur during pathological aging and aging‐related diseases such as AD.

